# Agency denied: PADA and the perils of paternalistic dog care

**DOI:** 10.3389/fpsyg.2026.1767922

**Published:** 2026-07-09

**Authors:** Sandra Foltin

**Affiliations:** Universität Duisburg-Essen, Essen, Germany

**Keywords:** animal assisted services, assessment, DAI, dog agency, dog assisted services, five freedoms, PADA, welfare

## Abstract

Dog-Assisted Interventions (DAI) or Dog-Assisted Services (DAS) are commonly regarded as compassionate practices, promoting human wellbeing through interspecies interactions. Recognition and popularity of adjunctive interventions and clinical applications have grown in recent decades. The increasing professionalization, whilst positive with respect to amplified knowledge in terms of the theoretical understanding and focus upon welfare, also poses risks, however, for the canine participants. Increasing classification to identify suitable animals by applying standardized tests and placing dogs into predetermined categories devalues their individuality, their idiosyncrasies, and their unique, authentic worth - the very qualities prized in dog-assisted interventions. This paper offers a critical analysis of the Personality Assessment for Dogs in Animal Assisted Interventions (PADA) — a test commonly utilized to evaluate dogs’ suitability for DAIs. The central objective is to evaluate the ethical and welfare implications of the PADA test design. While PADA describes itself as a safeguard for welfare, we come to the result that it produces the opposite effect: by emphasizing predictability, obedience and subservience, it risks undermining dogs’ agency, their emotional autonomy, and their genuine wellbeing, consistent with current positive welfare standards. Furthermore, every DAI/DAS relies on pro-active handler management. Competence, knowledge, mindfulness and attention to the individual dog’s strengths and limits and the commitment to protecting its welfare are the most essential factors determining the quality of any intervention. Only if the handler is empathetic, ensures suitable interaction conditions, and supports the dog through secure attachment - a quality that emerges within the relationship, shaped by the handler’s caregiving behavior and interaction style - can a DAI/DAS be considered truly effective and ethically responsible. Therefore, the core pre-requisite is the competent handler and any assessment must evaluate handler aptitude first. This critical component is entirely absent in PADA. A structured welfare audit of the PADA test was applied, using IAHAIO (International Association of Human-Animal Interaction Organizations) White Paper (2018/2023) ethical guidelines, Mellor’s Five Domains Model (2020) and the Agency-Based Welfare Science paradigm (Špinka, 2019) to examine how each exercise supports or restricts canine agency, autonomy, and affective engagement.

## 1 Introduction

In scientific life, it may be alluring to follow standardized procedures, but it is essential at times to pause and to consider whether these procedures are actually useful: Do they help or obscure our understanding of the being under observation? Domestic dogs exhibit substantial variation in their behavior, emotions and cognitive abilities (Horowitz, 2025). We measure behavioral and physiological responses and infer their meaning, knowing that our attempts to interpret behavior are vulnerable to subjectivity and a continuous influx of new information. Current theories regarding the mechanisms responsible for the therapeutic benefits of animal assisted interventions (AAI) agree on the idea that animals possess unique qualities that may facilitate and contribute to the success of a human therapy or offered service (Fine et al., 2025; Kruger and Serpell, 2010; Shoesmith et al., 2025), even though the exact “how” still remains elusive (Hediger et al., 2021).

Suitability assessments such as the PADA are typically conducted at an early stage of a dog’s prospective DAI/DAS career, in order to identify baseline behavioral tendencies, coping styles, and interaction pattern that are assumed to predict later performance. Important to note, that this early timing raises important conceptual and ethical considerations, as at this point in development, many, especially young dogs, are still undergoing cognitive, emotional, and social maturation, and their responses may be strongly influenced by transient factors such as novelty, environmental uncertainty, or the quality of the emerging handler–dog relationship. Minimum age requirements have been strongly recommended and are increasingly mandatory (ISAAT, 2026; Pet Partners, 2026; Tiergestützte Therapie Ausbildungszentrum NRW, 2026). Suitability for DAI/DAS may not be understood as a fixed trait identified at a single point of time, but rather as a dynamic, developmental process shaped through training, experience, and relational factors. The therapeutic benefits of dogs are frequently ascribed to their unique qualities. Dogs are perceived as non-judgmental, providing unconditional companionship and being emotionally attuned to the human. Studies suggests that these attributes facilitate emotional regulation, foster trust, decrease anxiety, and increase feelings of safety and comfort in therapy settings (Beetz et al., 2012; Crossman et al., 2015; Fine et al., 2025). Dogs’ natural ability to respond to human emotions may result in physiological changes, such as reduced cortisol levels and increased oxytocin in a person, both being associated with stress reduction and bonding (Guillén Guzmán et al., 2022; Kerepesi et al., 2015; Shoesmith et al., 2025).

Moreover, dogs offer alternative forms of communication, providing non-verbal assistance that may prove effective particularly well with recipients who have difficulty expressing themselves through words, such as those with autism or post-traumatic stress disorder (Barker et al., 2005). These qualities facilitate therapeutic outcomes by creating a safe, calming, and emotionally gratifying setting for recipients.

### 1.1 Dog assisted interventions/services

Dog-Assisted Interventions or DAS are structured, goal-oriented human–dog interactions with therapeutic, rehabilitative, educational, or recreational aims, involving the dog in an assisting role in order to improve the health and wellbeing of human recipients with physical, neurological, emotional and/or psychological disorders (Binder et al., 2024; Fine et al., 2025). DAIs can take different forms, including Dog-Assisted Therapy (DAT) — goal-directed therapeutic interventions delivered by health or human-services professionals — or Dog-Assisted Services (DAS) — more informal, motivational interactions without specific therapeutic goals (Fine et al., 2025; Kruger and Serpell, 2010; IAHAIO, 2023). DAI include a dog as facilitator to human services with the intention of improving health, educational, or psychosocial results of the recipients (Fine et al., 2025). Dogs are the animals most frequently utilized in settings and are an integral part of the DAI’s team (Bert et al., 2016; Horowitz, 2021; Santaniello et al., 2020), fulfilling a complex role tightly interlinked with the relational aspect of the human-animal bond.

### 1.2 Standardized tests

Standardized tests for therapy dogs are designed to evaluate a set of specific behaviors and traits that are deemed essential for all dogs, regardless of their individual personalities or temperaments in an intervention (Griffioen et al., 2023; Kawai et al., 2025; Sakurama et al., 2023). They aim to ensure consistency across evaluations. These tests typically measure factors like obedience, social behavior, and basic interactions with humans (Griffioen et al., 2023; Sakurama et al., 2023). For example, dogs are tested on their ability to sit, walk on a leash calmly, stay, or lie down, regardless of whether these tasks are in alignment with the dog’s natural disposition or the specific needs of the therapy environment (Sakurama et al., 2023; Stempiń and Strychalski, 2024). Different types of assessments are conducted to evaluate suitability for therapeutic work including behavioral assessments and emotional state evaluations. Behavioral assessments typically focus on observing the dog’s reactions to specific stimuli, their ability to interact with different people, and their response to various environments. Emotional state evaluations, on the other hand, assess how the dog is responding to stressors, how well it regulates emotions during sessions, and whether it displays signs of enjoyment or distress (Ameli et al., 2025; de Winkel et al., 2024).

### 1.3 The human handler of the team

The responsibility, the organization and all welfare provisions of any DAI/DAS setting lies, exclusively, with the handler; the dog merely serves as an “assistant.” The handler plays a pivotal role in guaranteeing animal welfare and the implementation of an ethical, dog-appropriate DAI/DAS. The handler must ensure a suitable stress management in degrees of severity (Ameli et al., 2025), and should have concrete ethological knowledge of the dog needs (Glenk and Foltin, 2021; Marinelli et al., 2009; Winkle et al., 2020) prior to, during, and after a DAI/DAS session (Ameli et al., 2025; Winkle et al., 2020). Safeguarding the appropriate stress management includes identifying and addressing varying levels and signs of stress and calming signals displayed by the dog: from mild phases of discomfort to more severe stress responses, including physiological reactions like panting or salivating (Winkle et al., 2020; Ameli et al., 2025) and modifying the intervention accordingly, to avoid overexertion and negative emotional states or trauma.

#### 1.3.1 Stress recognition

Studies indicate that many dog owners have limited abilities to recognize subtle signs of stress in their animal (Cortesi et al., 2025; Lind et al., 2017; Mariti et al., 2012), frequently overlooking nuanced indicators including gaze aversion, head turning, yawning and lip-licking (Mariti et al., 2012). Even trained observers have difficulty assessing stress, highlighting the complexity of accurate stress recognition (Lind et al., 2017; Kujala, 2017). Importantly, both acute and chronic stress can have significant welfare implications: Acute stress elicits measurable behavioral, cardiovascular, endocrine, gastrointestinal, and immunological changes, while chronic stress may lead to adaptive physiological alterations that mask ongoing distress (Beerda et al., 1997; Meyer and Forkman, 2014). Acute and chronic stress may have similar behavioral indicators, highlighting the importance of careful monitoring. A survey indicates that acute stress is relatively common during DAI/DAS, particularly with children, where unpredictable situations often trigger stress-related behaviors such as changes in posture, heightened alertness, altered facial expressions, and panting (Cortesi et al., 2025). Environmental stressors include high temperature, noise, transport to the setting, loud noises, unfamiliar environments and recipients and falling equipment (Cortesi et al., 2025; Glenk and Foltin, 2021). Cortesi et al. (2025) moreover postulate that predictability of environment and setting, workload, structured recovery and handler competence are critical components of welfare in DAI contexts, underscoring the necessity for handler education, systematic stress assessment, using multiple indicators and proactive removal and rest strategies to safeguard canine wellbeing.

Research on therapy dog welfare indicates that they may show signs of stress while handlers simultaneously perceive the experience as positive or enjoyable for the animal (Glenk, 2017; Winkle et al., 2020) revealing the incongruity between objective indicators and human interpretation (Hergovich et al., 2002; Zilcha-Mano et al., 2011a). This discrepancy is often attributed to anthropomorphic and optimism biases, where compliance or engagement is assumed to reflect enjoyment (Glenk, 2017; Glenk and Foltin, 2021; Townsend and Gee, 2021). Participation is typically human–initiated, with limited opportunities for dogs to opt-out. While canine behaviors my appear to be voluntary, the question of agency and voluntary consent remains complex, as dog participation is ultimately guided by its handler (Jones, 2025; Littlewood et al., 2023).

Ensuring animal welfare therefore requires precise planning, forward looking organization, adapting the setting if necessary, integration of relaxation phases and the continuous use of positive reinforcement by the handler. The handler must guarantee a place of retreat, enabling free encounters, safeguarding the dog’s agency while paying close attention to its physiological and psychological needs (de Winkel et al., 2024; Jones, 2025).

#### 1.3.2 Attachment style and relational reciprocity

The relational dimension of the human–dog team is essential for an effective intervention (Menna et al., 2019; Payne et al., 2015; Zilcha-Mano et al., 2011a) and attachment factors strongly affect the performance of the dog (Foltin and Ganslosser, 2022). Dogs with a secure attachment style are more likely to exhibit cooperative behaviors, attentiveness, eye contact, optimism and engagement - all essential elements of positive therapeutic results (Menna et al., 2019; Sobol and Dowling-Guyer, 2026; Walsh et al., 2025). For a secure attachment style, the dog–handler relationship should be based on trust, reliability, non-aversive handling and training. Dogs respond differently to different persons depending on familiarity (Pinelli et al., 2026; Horn et al., 2013; Jamieson et al., 2018a; Zilcha-Mano et al., 2012) and the relationship directly affects the team’s quality of interaction (Jamieson et al., 2018a,b).

Attachment style and relational reciprocity also affect the complexity of an intervention (Cox, 2025; de Santis et al., 2024; Mair et al., 2025). Relational reciprocity as well as attachment style impact the quality and dynamics of the interaction within the intervention, affecting responsiveness, coordination and the ability of the dog-handler team to engage appropriately with the recipient.

A secure attachment to the handler decreases the animals’ stress level (de Souza et al., 2025; Martin et al., 2025; Schöberl et al., 2016). In addition, social support and escape/opt-in/opt-out options, such as allowing the dog to take breaks, withdraw or leave the setting, further mitigate stress and heighten welfare parameters (Jones, 2025).

Therefore, handler and dog should be trained, educated and qualified together because a balanced, responsive relationship is vital for ensuring the dog’s welfare and optimizing the intervention’s success (Erichsmeier et al., 2025; Payne et al., 2015; Zilcha-Mano et al., 2011b). It has been postulated that the ideal setting is a single dog and a single handler as a team with the handler having a strong emotional bond to the dog (Erichsmeier et al., 2025; Nolan and Gravitte, 1977; Tomkins et al., 2011).

### 1.4 One health and DAI/DAS

By definition, DAI/DAS require handler and dog to act in concert. From a One Welfare perspective, the offered services should consequently not only improve the health of recipients, but also safeguard the wellbeing of dog and handler (Chalmers and Dell, 2015; Glenk and Foltin, 2021; Hediger et al., 2019; Horowitz, 2021).

The One Health concept (Destoumieux-Garzón et al., 2018; Pitt and Gunn, 2024) refers to the interconnectedness of human and non-human animals and their environment, pointing out that the wellbeing of each is intrinsically linked. In the context of DAI/DAS the One Health concept is particularly relevant because the interventions rely on the complex relationship between humans and dog where the wellbeing is considered in tandem within a triadic setting. The effectiveness of the intervention depends not only on the physical and emotional state of the dog, but also on the dynamics between dog, handler and recipient. Factors like attachment style, personality traits and training methods significantly influence the outcomes of the intervention (Casey et al., 2021; Fernandes et al., 2017; Mair et al., 2025; Wanser and Udell, 2019). One Health calls for a holistic method to evaluate DAI/DAS, considering the full spectrum of factors contributing to success and wellbeing (Hediger et al., 2019). Standardized tests, which evaluate dogs based on uniform criteria without accounting for these unique, relational elements, fail to capture the depth of interspecific relationships. Individualized assessments that consider emotional states, personalities and specific interactions between dog, handler and recipient are more aligned with the One Health concept, ensuring that emotional and physiological aspects of the individuals involved are optimized (Chalmers and Dell, 2015; Menna et al., 2019).

### 1.5 Animal welfare frameworks

Animal welfare science has progressed from preventing animals from “suffering” toward promoting positive affective states and agency (Binder et al., 2024). Traditional frameworks such as the “Five Freedoms” provide an indispensable foundation for safeguarding animals from harm (Fine and Griffin, 2022; Mellor, 2015; Peralta and Fine, 2021). Mellor’s “Five Provisions and Welfare Aims” reframe welfare assessment, aligning with the “duty of care” clauses found in Section 10 (S10) of Animal Welfare Acts internationally (e.g., UK Animal Welfare Act 2006) (Horton, 2024), New Zealand Animal Welfare Act 1999 (Robertson and Goldsworthy, 1999). These provisions translate legal obligations—warranting appropriate nutrition, an enriching environment, freedom of behavioral expression, and protection from harm—into actionable welfare standards (Horton, 2024; McCulloch, 2019).

The ethical framework provided by the IAHAIO guidelines (International Association of Human Animal Interaction Organisation [IAHAIO], 2023) was selected as one central reference point for the analysis herein, because it represents a most comprehensive and internationally recognized standard, specifically developed for AAIs. The IAHAIO guidelines are explicitly tailored to the unique context of human-animal interactions including DAI/DAS, integrating key principles from contemporary welfare science, such as the promotion of positive affective states, the safeguarding of agency, and the requirement of voluntary participation. As such, they provide a context-sensitive benchmark that reflects both, ethical obligations and practical considerations relevant to DAI/DAS. In this paper, the IAHAIO framework is not treated as an unquestioned authority, but as a guiding ethical reference point against which the PADA assessment can be systematically evaluated. Moreover, using IAHAIO guidelines alongside complementary models – such as the Five Domains Model and Agency-based Welfare science – enables a multidimensional assessment.

For DAI/DAS, frameworks such as the IAHAIO Guidelines (2023 update) and the Five Domains Model (Mellor et al., 2020) emphasize agency, and the animal’s right to exert choice and control over its participation. Jones’ (2024) “Canine Consent” model augments this principle by operationalizing consent and voluntary engagement in canine interactions, stating that welfare integrity depends on the dog’s constant ability to opt-in or out of activities. Consent mechanisms are observable procedures that allow the dog to actively indicate willingness to engage (opt-in) or disengage (opt-out) without coercion or penalty, such as offering approach opportunities, respecting avoidance or withdrawal behaviors, and incorporating predefined stop or termination criteria (e.g., an exit target).

Špinka (2019) moreover differentiates different stages of animal agency. He describes four levels from low to high, reflecting the degree to which an animal actively controls and influences its own actions and environment. At the lowest level, agency involves simple automatic responses to stimuli – the dog reacts in relatively fixed ways, with reduced flexibility or control. The second level includes goal-directed behavior, where the dog actively performs actions to achieve a specific outcome, such as obtaining food or avoiding discomfort. The third level involves choice and behavioral flexibility: The dog may select and elect among several possible actions. It has context dependent choices, evaluating options and adjusting behavior accordingly. The fourth and highest agency level includes complex, self-initiated and self-determined, socially informed behavior. The dog deliberately and with free choice influences its environment, learns from experience and may coordinate actions with others, reflecting a high degree of control, empowerment and self-reliance.

Špinka (2019) posits that complex agency levels increase subjective experience including existential requirements like exploring, making choices and having control and autonomy. Within this line Rault et al. (2025) provide the following definition of welfare: “Positive animal welfare is defined as the animal flourishing through the experience of predominantly positive mental states and the development of competence and resilience” including positive affect, control over actions and environment, as well as opportunities to make choices and pursue goals through ensuring agency.

Together, these models converge on a shared goal: to guarantee and facilitate a positive, self-determined, meaningful interaction for the dog and not merely the absence of suffering.

## 2 The PADA assessment tool

The PADA (PADA, 2025) was created to evaluate a dog’s suitability for participation in DAI/DAS. It was developed collaboratively by the Animals for People Association (Poland), the International Center of Anthrozoology ICofA (Norway), and Eötvös Loránd University (Hungary) as a standardized assessment tool designed to evaluate various personality traits and behaviors in dogs that are deemed important for their roles in DAI/DAS. The ICofA is the central organizer of the PADA protocol, coordinating PADA-ICofA, the standardized animal personality assessment system. It is responsible for certifying PADA evaluators, collecting registration fees for test results, maintaining guidelines and overseeing the international registry (“map”) of certified evaluators. ICofA offers a certification course to become a “PADA ICofA Evaluator” and administers a certification course for those interested in becoming PADA evaluators. After completion, individuals are certified and able to conduct evaluations.

While PADA is a standardized assessment tool, it operates similarly to a franchise system with evaluators’ charging fees for individual evaluations. The cost for testing a dog through PADA typically ranges from €180–200, with evaluations for cats available as well (€175–205). The certification course for potential evaluators costs approximately €1,649 (PADA Poland, 2025). Details on the PADA system and evaluator network are available through the official website.^1^

Limited research has examined how the PADA’s structure and techniques fit within contemporary welfare frameworks, emphasizing positive experiences, animal agency, and consent. This paper addresses these gaps by systematically comparing the 18 PADA assessment exercises^2^ with the welfare standards proposed by IAHAIO guidelines (2018/2023), Mellor’s Five Provisions and Welfare Aims (2020), Spinka’s levels of agency (2019), and Jones’s Canine Consent framework (2024). These frameworks are crucial for understanding whether the PADA exercises align with contemporary ethical standards. The original 18 exercises may be found in the Supplement.

## 3 Objectives of the comparative analysis

The goals of the analysis are to

1. Evaluate each of the 18 PADA test situations against the aforementioned frameworks to assess if they meet, partially meet, or fail to meet the required standards
2. Identify potential welfare risks or stressors inherent in PADA procedures; and
3. Propose evidence-based modifications to improve PADA’s alignment with international welfare standards.

By conducting this comparative analysis, the paper aims to provide recommendations that will improve the PADA assessment tool and ensure its ethical application. Further, it purports to offer a continued refinement of all current assessment tools for dogs in DAI/DAS, promoting not only safe participation but also wellbeing grounded in scientific and ethical principles.

### 3.1 Key PADA exercises

The PADA test includes a range of standardized exercises (*n* = 18) designed to assess various aspects of dogs’ behavior and temperament along three interrelated dimensions: social interaction, stress response, and attention and focus:

1. Social interaction test: In these exercises, the dogs’ response to human interaction is evaluated by observing how it engages with unfamiliar people. The dog must exhibit positive social conduct such as approachability and comfort with handling to pass this exercise.
2. Stress response test: These exercises expose the dog to stressors such as loud noise to assess how well the dog manages stress and whether it displays distress. Dogs that remain calm and adaptive are considered more suitable for DAI/DAS.
3. Attention and focus test: In these exercises, the dog must follow commands and display the ability to maintain focused amid distractions to pass/to be considered as suitable for DAI/DAS.

#### 3.1.1 Evaluation of PADA exercises using welfare frameworks

Each of the 18 PADA exercises was assessed across three integrated dimensions: IAHAIO compliance, Mellor’s Five Domains of Animal Welfare, and Špinka’s levels of agency. These dimensions are essential for understanding how PADA exercises support or restrict canine agency, autonomy, and emotional engagement.

1. IAHAIO compliance audit: Point-by-point comparison of each exercise was evaluated for alignment with IAHAIO’s ethical guidelines, particularly those related to animal welfare and participation (complete list, see Supplementary materials).
2. Mellor’s five domains: This model identifies five welfare domains: nutrition, physical environment, health, behavioral interactions, and mental state. We focused particularly on Domains four (Behavioral Interactions) and five (Mental State), as these are most likely to be affected in the context of the PADA assessment (complete list, see Supplementary materials).
3. Agency level classification: Each exercise was classified according to Špinka’s hierarchy of agency, which ranges from passive/reactive agency (where the dog is controlled by the handler), to aspirational agency (where the dog is intrinsically motivated and engaged, for details, see Supplementary materials).

#### 3.1.2 Scoring and rating system

Each exercise received:

1. IAHAIO compliance score
  - Fail (contradicts IAHAIO standards).
  - Moderate = partial compliance but contains moderate welfare risks.
  - Full compliance.
2. Mellor domain impact
  - Each situation was mapped onto the Five Domains Model to identify potential risks in physical, behavioral, or affective domains.
3. Agency level
  - The degree of agency was coded according to Špinka’s (2019) hierarchy (see Supplementary materials).

**FIGURE 1:**
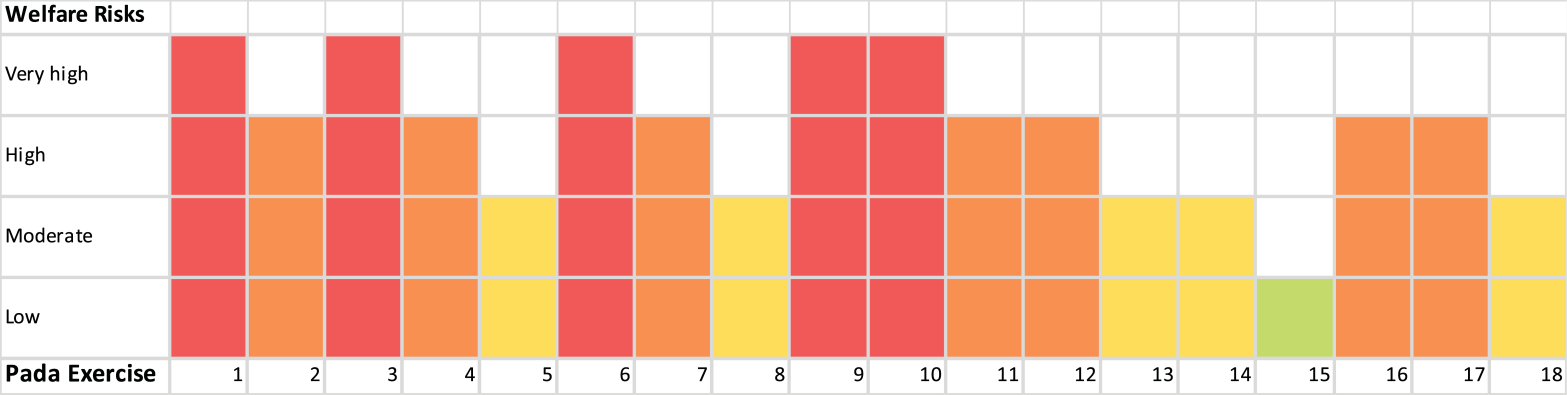
Summary of the welfare risks (low–very high) for each (1–18) Personality Assessment for Dogs in Animal Assisted Interventions (PADA) exercise.

Overall welfare risk rating — Each exercise received an overall risk rating (Low, Moderate, High, Very High), with behavioral justifications for higher-risk exercises (e.g., suppression of natural behaviors), see Figure 1. For exercises rated moderate to very high, modifications are proposed to improve compliance with IAHAIO guidelines and welfare standards.

### 3.2 Scoring matrix and data synthesis

Table 1 summarizes each exercise with its risk rating, violated principles, and affected domains. The table encompasses the following categories:

**TABLE 1:** Each Personality Assessment for Dogs in Animal Assisted Interventions (PADA) exercise (1–18) assessed against International Association of Human-Animal Interaction Organizations (IAHAIO) compliance; welfare risk; agency level; Mellor’s domains impacted; key violations/concerns and recommended IAHAIO-compliant revisions.

PADA exercise	PADA purpose	IAHAIO compliance and violations	Welfare risk	Agency level (Špinka, 2019; Rault et al., 2025)	Mellor domains impacted	Key violations/concerns	Recommended IAHAIO-compliant revision
1. Entering New Environment with Stranger and Dog	Tests reactions to other dog and cooperation with handler	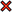 Fails. Violates sections 4.5, 4.1, 3.5, 3.2, 2.3 Req. 1, Req. 5	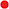 High→ 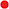 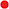 Very high (context-dependent: corridor width, noise).	1 = Passive; no agency	2, 4, 5	Forced proximity, no choice to withdraw, sensory overload; stress signals unmonitored, no prior exploration, no free movement - dog is on a leash, no option to retreat: dog cannot choose safe individual distance and may suppress signals (freezing). May lead to attachment problems with handler if dog feels not protected.	Dog should be off-leash. Allow exploration time and voluntary approach, gradual acclimatization; implement stress-based stop rule, splitting, management of the handler essential. Observers monitor stress signals and stop or modify to ensure welfare. Train handler in welfare indicators and responsibility of dog-friendly management.
2. Interaction with stranger	Measures sociability toward unfamiliar person and tactile tolerance	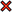 Fails. Violates sections 4.1, 3.5, 3.2, 2.3 Req. 1, Req. 5	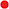 High	1 = Passive → action-driven only if consent cues used; no agency	2, 4, 5	Stranger-initiated touch without consent of the dog, risk of hidden distress, lack of retreat option, no stop rule, no opt-in/opt-out choice; selection bias toward tolerant-but-stressed dogs.	Introduce consent-based approach (voluntary contact ≥ 2 s) of the dog, choice of distance and duration; trained welfare monitor; allow withdrawal; handler management essential, reassurance permitted. Train handler in welfare indicators and responsibility of dog-friendly management.
3. Rough handling/microchip simulation	Tests tolerance of invasive and rough handling	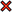 Fails. Violates sections 4.5, 4.1, 3.5, 3.4, 3.2, 2.3	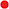 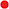 Very high	1 = Passive/reactive; no agency	2, 3, 4, 5	Deliberate distress induction; no opt-out choice. Intentional roughness and invasive handling of sensitive areas may cause negative association through learning experience and may cause situational generalization. May cause pain, startle and /or traumatize the dog and lead to learned helplessness. Prolonged restraint; discomfort; absence of welfare-trained observer. May lead to attachment problems with handler if dog feels not protected. Disproportionate exercise.	Replace with cooperative handling protocol; habituate equipment. Limit handling to essential veterinary contexts. Prior medical training mandatory; stop at first avoidance cue; train evaluators in welfare indicators. Train handler in welfare indicators and responsibility of dog-friendly management.
4. Calling the dog	Measures sociability after high stressors	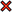 Fails. Violates sections 4.6, 4.1; 3.4, 3.2, 2.3	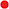 High	1 = Reactive; no agency	3, 4, 5	Testing re-approach immediately after a high, potentially traumatizing stressor. Risk of appeasement signals instead of voluntary sociability; coerced recall. Selection bias toward tolerant-but-stressed dogs. No stop rule, no opt-in/opt-out choice, no recovery-period.	Include recovery period; acclimatization, voluntary recall. Introduce consent-based approach (voluntary contact ≥ 2 s) of the dog, choice of distance, duration and gentle invitation. Observers monitor stress signals and stop or modify if needed. Train handler in welfare indicators and responsibility to say no.
5. Walking into a new room	Observes exploratory behavior	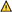 Partial Violates sections 4.5, 4.4, 4.1, 3.5, 3.2, 2.3	?? Low to moderate	1 = Reactive; no agency	2, 4, 5	Ambiguity in handler role; limited time; incomplete welfare safeguards.	Extend acclimatization time; permit voluntary exploration; define intervention criteria. Observer monitor stress signals and stop or modify if needed to ensure welfare. Train handler in welfare indicators and responsibility of dog-friendly management.
6. Exposure to sudden noise/scream test	Tolerance of unpredictable behavior and emotions	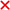 Fails. Violates sections 4.5, 4.1, 3.4, 3.2, 2.3	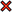 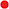 Very high	1 = Passive/reactive; no agency	2, 3, 4, 5	Startle reflex unavoidable; recovery time not defined; risk of over-exposure and potential trauma; No opt-out; observer criteria ambiguous. Produces acute physiological distress and may cause prolonged hypervigilance and trauma. Without opt-out or habituation this is disproportionate. May lead to attachment problems with handler if dog feels not protected.	Use graduated auditory exposure; permit voluntary approach (opt-in/opt-out); record latency to recover; end test if avoidance > 2 s; provide decompression break. Train handler in welfare indicators and responsibility of dog-friendly management.
7. Knocking on the door	Measure guarding resources and territory, response to strange noise + stranger approach	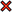 Fails. Violates sections 4.5, 4.1, 3.4, 3.2, 2.3	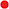 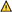 Moderate → High (if repeated or intense).	1–2 = Action-driven; no agency	2, 4, 5	Deliberate stress induction; no opt-out choice. Abrupt knocks, after prior high stressors, may produce cumulative negative effects and distress. Negative association through learning experience, may cause situational generalization. Ambiguity about “intense” knock leaves scope for excessive startle. Forced exposure. Without opt-out or habituation this is disproportionate. Observer criteria ambiguous.	Gradual knock; allow retreat; voluntary stranger interaction; structured reinforcement; observer intervention. Prior auditory enrichment training. Optional greeting only if dog approaches. Observer monitor stress signals and stop or modify if needed. Ensure welfare oversight. Train handler in welfare indicators and responsibility of dog-friendly management.
8. Dog’s interest toward humans when called	Evaluate voluntary approach/sociability after stressful situation	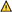 Partial. Violates sections 4.5, 4.1, 3.5, 3.2, 2.3	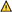 Moderate (can be higher after stress)	1–2 = Action-driven; no agency	2, 3, 4, 5	Repeated calling limits agency; cumulative stress; opt-out absent; approach may be coerced.	Single gentle invitation; handler support; acclimatization, voluntary approach; mark “declined – choice respected”; positive reinforcement. Observer monitor stress signals and stop or modify if needed. Train handler in welfare indicators and responsibility of dog-friendly management.
9a. Wheel-chair (ghost)	Strategy to cope with fear. Assess coping with ambiguous, potentially highly frightening stimulus in dark corridor	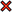 Fails. Violates sections 4.5, 4.4, 4.1, 3.2, 2.3	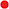 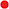 Very high	1 = Passive/reactive; no agency	2, 3, 4, 5	Ambiguous covered human in dark corridor is a strong fear-provoking stimulus; leash compounds inability to retreat. Repeated forced passes risk learned avoidance/helplessness, cumulative stressors. Negative association through learning experience, may cause situational generalization and trauma. Forced exposure. No opt-out. May lead to attachment problems with handler if dog feels not protected. Disproportionate exercise.	Dog is off the leash, gradual exposure, acclimatization. Appropriate setting, voluntary approach and distance. Time to explore with all senses, safe retreat, observer-monitored stress stop, positive reinforcement. Train handler in welfare indicators. Explicit instruction to stop if dog shows distress.
9b. Wheel-chair walk and habituation	Habituation to fearful stressors. Repeated exposure to moving, covered human	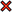 Fails. Violates sections 4.5, 4.4, 4.1, 3.2, 2.3	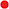 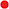 Very high	1 = Passive/reactive; no agency	2, 3, 4, 5	Repeated back-and-forth passes by ambiguous, covered human in dark corridor is a strong fear-provoking stimulus; leash compounds inability to retreat. Repeated forced passes risk learned avoidance/helplessness and trauma. Cumulative stressors. Negative association through learning experience, may cause situational generalization and learned helplessness. Forced exposure. No opt-out. May lead to attachment problems with handler if dog feels not protected. Disproportionate exercise.	Dog is off the leash. Check if dog voluntarily enters situation/corridor again. Limit passes, only voluntary interaction, gradual, voluntary approach, observer-based stop, opt-in/out regardless of proximity. Explicit instruction to stop if dog shows distress. Train handler in welfare indicators and responsibility of dog-friendly management.
10. Crutches	Evaluate boldness: coping strategies with moving, unusual object/ human in dark corridor, habituate to strange walk (due to crutches), noise sensitivity	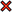 Fails. Violates sections 4.1, 3.2, 2.3	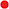 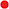 Very high	1 = Passive/reactive; no agency	2, 3, 4, 5	Commanded movement, no opt-in/out choice, leash restriction, repeated forced exposure, risk of learned avoidance/helplessness and trauma, ambiguous stimulus, constrained space, novel and potentially startling and/or traumatizing situation; repeated exposure without choice risks of cumulative stress. May lead to attachment problems with handler if dog feels not protected. Disproportionate exercise.	Dog is off the leash. Voluntary entry, exploration, gradual exposure, voluntary choice of duration and distance, re-assurance of handler, optional investigation, observer-based stop, positive reinforcement. Monitor stress signals and stop or modify if needed. Ensure welfare oversight.
11. Jump on a bed	Assess boldness, sociability, socialization and willingness to interact with stranger	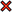 Fails. Violates sections 4.5, 4.1, 3.2, 2.3	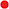 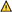 Moderate-high	1–2 = Action-driven if voluntary; passive when commanded In that case no agency.	2, 3, 4, 5	Forced obedience, dog is on a leash and restricted, contingent rewards, physical risk, repeated prompts. Requirement to jump may create physical hazard and emotional pressure; contingent petting/rewarding on obedience may be coercive as it relates to asymmetries of control, dependency on human-mediated resources and the potential restriction of behavioral autonomy within a structured test environment. Concerns arise in this context when reinforcement delivery is embedded within rigid obedience demands that may constrain voluntary engagement or discourage the expression of avoidance or discomfort signals.	Dog is off the leash. Voluntary choice, prior experience, enrichment/training, safe stable bed, minimal prompts, unconditional positive interaction, observer-monitored stress. Train handler in welfare indicators and responsibility of safe and dog-friendly management.
12. Stairs with examiner	Assess cooperation with stranger, boldness, social skill, socialization and physical confidence on stairs	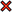 Fails. Violates sections 4.1, 3.5, 3.2, 2.3	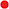 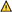 Moderate-high	1 = Passive/reactive under leash transfer; no agency	2,3,4,5	Leash transfer, forced obedience, stair-related physical risk, repeated coaxing may induce anxiety; mobility-challenged dogs at risk. Attachment issues may be raised by having to leave its handler. In conflict with rule that handler never leaves the dog unattended or with third person. Risk of appeasement signals instead of voluntary sociability; coerced recall. Selection bias toward tolerant-but-stressed dogs. May lead to attachment problems with handler if dog feels not protected. No stop rule, no opt-in/opt-out choice.	Handler retains leash and stays with the dog at all times, one invitation only, safe stairs, optional refusal, positive reinforcement, observer-monitored stress. Prior stairs training and third party taking the leash training, while the handler is present at all times.
13. Sit	Evaluate short-term obedience and coping after prior stressors	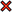 Fails. Violates sections 4.5, 4.1, 2.3	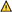 Moderate	1 = Passive/reactive; no agency	4,5	Forced repeated commands, reduced choice, potential mental stress accumulation	Voluntary engagement, minimal prompting, one gentle invitation, immediate release, unconditional reinforcement, observer and handler stress monitoring.
14. Lie down	Assess obedience, coping, and retention in position	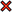 Fails. Violates sections 4.5, 4.1, 2.3	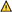 Moderate	1 = Passive /reactive; no agency	4,5	Forced repeated commands, potential mental distress and reduced agency. Same issues as sit — repeated coercion undermines welfare if dog is tired or stressed.	Voluntary engagement, one gentle prompt, immediate release if stressed, unconditional reinforcement, observer monitoring.
15. Stand up	Evaluate response to handler release cue and short-term obedience	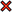 Fails. Violates sections 4.5, 4.1, 2.3	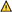 Low–moderate	1 = Passive/ reactive; no agency	4,5	Repeated cues, limited choice, mild mental stress	Voluntary choice, one gentle prompt, unconditional reinforcement, observer-monitored stress intervention.
16. Crowd	Assess boldness, sociability and composure under multiple approaching strangers	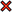 Fails. Violates sections 4.5, 4.1, 3.2, 2.3	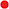 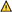 Moderate–high	1 = Passive/reactive; no agency	2,4,5	Forced proximity, leash restricts avoidance and exacerbates distress. No choice of distance and/or duration. No opt-in/opt-out choice. Individual or breed differences ignored. Crowd simulations can overwhelm dogs, no escape options.	Dog is off the leash. Voluntary proximity and duration, limit number of approaches and number of people, allow retreat, opt-in/opt-out choices, observer time-monitored termination. Train handler in welfare indicators and responsibility of safe and dog-friendly management.
17. Food	Assess resource defense, tolerance and coping with removal of food	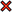 Fails. Violates sections 4.5, 4.1, 3.5, 3.2, 2.3	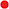 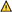 Moderate–high	1 = Passive/ reactive; no agency	1,3,4,5	Forced tolerance of food removal, risk of frustration or resource guarding, limited choice, can provoke stress or aggression, especially subsequent to prior stressful exercises.	Voluntary engagement with food, no frustration creation, non-coercive handling, positive reinforcement regardless of compliance, observer intervention for guarding or stress, respect individual differences.
18. Toy play with stranger	Assess if dog can play with stranger after post-stressors	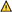 Partial violation Sections 4.5, 4.1., 3.5, 2.3	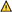 Low–moderate	1 = Passive/ reactive; no agency	4,5	Positive engagement if voluntary; some dogs do not play with toys, repeated encouragement after fatigue or stress can negate benefits, opportunity for exploration; minimal welfare concern if handler respects signals.	Maintain voluntary participation; rotate toys for novelty; include rest interval; use this exercise as welfare-positive counterbalance. Dog may not want to play; needs freedom to disengage. Observers monitor stress signals and stop or modify.

1. PADA Test Situation (1–18)
2. PADA purpose (as stated in the PADA manuscript)
3. IAHAIO Compliance Rating (Meets/Partial/Fails)
4. Welfare Risk Level (Low/Moderate/High/Very High)
5. Agency Level (Passive, Action-driven, Competence-building, Aspirational)
6. Affected Domains (1–5)
7. Key violations/concerns
8. Recommended IAHAIO-compliant modifications

A scoring matrix summarizes each exercise’s welfare risks, compliance ratings, and recommended modifications. Across test components, patterns are analyzed to identify systemic welfare deficits—particularly those arising from emotional suppression, loss of agency, or one-directional human benefit.

#### 3.2.1 Example of PADA exercises

Personality Assessment for Dogs in Animal Assisted Interventions exercise 1: Dog and handler enter a new environment (inside: a hall, a room) with a stranger and an unknown dog. Both dogs are leashed. The dogs must pass each other. Purpose: test social skills; cooperation with handler; dog’s aptitude in novel situations and with unknown dog(s).

Personality Assessment for Dogs in Animal Assisted Interventions exercise 1 was evaluated and found to violate five IAHAIO Sections (§§ 4.5, 4.1, 3.5, 3.2, 2.3 Req. 1, Req. for details see Table 1).

Ethical concerns (consent and agency): The dog is required to enter an unfamiliar environment and approach an unfamiliar dog. Both are leashed. The dog has no meaningful opportunity to opt-out or increase his distance. Because the handler directs movement (“go where they are asked to go”), compliance may reflect training pressure rather than voluntary participation. Forced proximity to a potential social stressor without choice can compromise autonomy and does not reflect the dogs’ preferences or natural behavior.

Behavioral concerns: Dogs typically display subtle stress and calming signals in a dog-dog situation (head turned, eyes lowered, slow movement, lip licking). Evaluators and handler may misinterpret these as calmness rather than coping strategies. If the dog is prevented from normal social distancing (curved approach, avoidance arc) the situation may suppress communication signals, increasing risks of escalation. Repeated exposure may lead to negative associations with other dogs or new environments or even to learned helplessness.

Physiological concerns: Novel environments plus social uncertainty activate arousal systems (sympathetic nervous system and HPA axis) and elevate cortisol levels. Chronic repetition without adequate recovery periods may affect the emotional regulation, increase vigilance and bias learning toward threat sensitivity. Leash restraint can heighten reactivity through frustration and reduced perceived control. If the handler uses tension on the leash, verbal correction or pressure, the dog may associate the handler with social stress, potentially reducing trust and increase anticipatory anxiety in future settings.

Result: This exercise did not adequately provide the dog with a right to withdraw or opt-out. Additionally, the exercise did not take into account the potential for emotional distress when the dog is placed in a novel, potentially overwhelming situation without the option for retreat. The dog is not offered time to explore prior to the test situation. Based on these observations, recommended modifications are to allow the dog to explore, disengage (exit/opt-out) and recover if necessary.

### 3.3 Ethical positioning

This analysis is grounded in an ethics-of-care and positive welfare perspective (Donovan, 2022; Mellor et al., 2020; Rault et al., 2025), treating the dog as a sentient associate rather than an instrumental tool. Welfare integrity is defined by the manifestation of agency, awareness, and positive affective engagement of the dog, not solely by the absence of harm.

## 4 Findings

Overall, the analysis indicates systematic welfare and ethical concerns across the assessed PADA exercises. As summarized in Figure 1 and Table 1, the majority of tasks suppress canine agency, rely on distress-based or ambiguous stimuli, and lack adequate welfare monitoring procedures. None of the 18 exercises meets full compliance with IAHAIO standards, while only four to five tasks may be considered conditionally compliant, if implemented with voluntary engagement and low affective load. The concerns identified correspond closely with previously reported risks regarding passive role assignment and welfare compromise in assessment contexts (Špinka, 2019; Jones, 2025).

### 4.1 PADA analysis

Across the 18 PADA tasks, four deficits consistently emerged. The majority of exercises (see Figure 1 and Table 1) show clear or probable violations of §§4.6, 4.4, 4.1, 3.4, 3.5, and 2.3. These include the absence of voluntary participation mechanisms (IAHAIO §4.1), lack of predefined stop or termination criteria (§2.3), insufficient respect for dog communication signals such as avoidance or calming behaviors (§3.5), use of stress-inducing stimuli without proportional justification (§3.4), and omission of habituation, recovery, and predictability measures (§§4.4, 4.6).

#### 4.1.1 Canine agency

First, canine agency is routinely suppressed, with exercises preferring compliance and subjugation to voluntary social engagement (see Figure 1 and Table 1). This contravenes IAHAIO §4.1 on voluntary participation and the right to withdraw, by omitting explicit voluntary opt-out/opt-in mechanisms (Jones, 2025). With regard to the handler, clear stop/termination criteria are completely lacking (§2.3), and a number of stressors are used without proportional justification (§3.4). For instance startle-based stimuli (e.g., sudden noises or unexpected object movements), exposure to ambiguous or threatening human approaches, forced handling or restraint, navigation of unfamiliar or constrained physical environments (e.g., stairs or narrow corridors), and resource-related challenges. These stressors may induce elevated arousal or distress without clear relevance to the intended competencies for DAI/DAS suitability, thereby raising proportionality concerns under positive welfare guidance.

The majority of exercises violate respect for dog-specific signals (stress signals/calming signals § 3.5) and lack of emphasis on positive, affiliative engagement (§4.5). Many exercises prioritize obedience or tolerance under pressure rather than reinforcing voluntary social interaction, cooperative behavior, or positive affective states. Examples include limited opportunities for rewards-based engagement, minimal encouragement of affiliative contact initiated by the dog, and task structures that emphasize compliance despite uncertainty or distress, rather than mutually reinforcing interactions between dog and handler.

Structured habituation phases and formalized recovery intervals between exercises are either absent or insufficiently specified in the current PADA protocol (as applicable to the documented version analyzed). This lack of operational guidance limits the capacity to ensure controlled exposure, the prevention of cumulative stress effects across successive test components and emotional recovery of the dog. Habituation or recovery periods appear to depend on individual evaluator implementation rather than being systematically required by the protocol.

Habituation procedures, mandatory recovery, predictability and debriefing measures are missing (§§ 4.4, 4.6). The application of Špinka’s hierarchy suggests that the assessed tasks predominantly position dogs in passive or reactive roles (Table 1).

#### 4.1.2 Conditions based on distress-based stimuli

Second, conditions based on distress-based stimuli - particularly in exercises involving startle responses, ambiguous human figures or forced handling (see Table 1) - conflict with the positive welfare paradigm (Englund and Cronin, 2023; Littlewood et al., 2023; Mellor, 2015). Mellor’s (2020) Domains 4 (behavioral interactions/expression) and 5 (affective or mental state) were the most consistently affected, because the majority of exercises involved constraints on the dog’s ability to express species-typical or self-regulated behavior, combined with exposure to stimuli likely to induce negative affective states such as uncertainty, fear, or distress. In contrast, impacts on Domains 1–3 were more task-dependent and occurred primarily in exercises involving physical challenges or handling components.

#### 4.1.3 Welfare monitoring procedures

Third, welfare-monitoring procedures are insufficient. The absence of predefined stop or termination criteria suggests that decisions to interrupt or adjust testing procedures are left largely to the subjective judgment of the observer. While observer monitoring of stress signals is generally expected as part of the professional competence, it is not currently operationalized through standardized objective stop rules within the PADA protocol (as applicable to the documented version analyzed). These omissions fail compliance with IAHAIO §§2.3 and 4.6 and risk aggregating cumulative distress. Mellor’s Domains analysis similarly proposes that besides Domains 4 (Behavioral Interactions) and 5 (Mental State), physical environment (Domain 2) and functional state (Domain 3) are impacted in tasks involving stairs, tight corridors, handling, or resource management. Notably, these exercises carry elevated risks, consistent with cumulative effects of affective carryover, fatigue and novelty.

Personality Assessment for Dogs in Animal Assisted Interventions testing procedures select dogs that tolerate aversive stimuli rather than those that prefer human interaction — an ethical and validity problem for DAI/DAS suitability.

An important dimension that warrants consideration and discussion is the potential safety implication of not systematically and transparently evaluating dogs participating in DAI/DAS: The dog’s responses to aversive or highly salient stimuli like the exposure to unpredictable human behavior, sudden environmental changes, loud noises, or intrusive contact carry risk, like startle responses, or in rare cases, defensive behavior. If such response pattern are not adequately assessed under controlled and ethically managed conditions during pre-interaction procedures, there is a risk that dogs may be placed in settings for which their coping strategies are insufficient or not yet reliably expressed.

However, a welfare conform assessment can also evaluate dogs prior to being placed in an intervention. If procedures are embedded within a framework ensuring predictability, and the possibility to withdraw, coping capacities can be evaluated without the use of highly aversive stimuli. Instead tests may rely on thorough preparation of both the handler and the dog, including graded exposure, foreseeable working conditions and the establishment of a secure dyadic relationship prior to testing. Safety does not depend on inducing fear responses but can be ensured through handler responsibility, careful environmental design and prior positive habituation to relevant stimuli.

#### 4.1.4 Handler participation

Fourth, handlers participating in the test should be assessed prior, to determine their ability to accurately read their dog’s communication signals, including avoidance and calming signals, fear and stress behavior. Handlers should be obliged to demonstrate an understanding of humane training principles, learning techniques, attachment theory and the One Health concept. Handler training methods – including aversive techniques - must be disclosed and evaluated. Participation requires the handler to recognize the dog as a team partner and Animal welfare/rights must be placed above performance outcomes. These principles should be discussed, verified, and reflected upon with the handler, preceding the test.

All exercises should be described to enable handler to recognize potential physical and/or mental risks to their dog’s welfare. Handlers should be informed that they might stop an exercise at any time without penalty. The handler’s primary duty lies in protecting their dog, ensuring humane treatment, appropriate stress management and compliance with current welfare standards at all times. *Kadavergehorsam* (“blind or rigid obedience”) of human or dog should never be the purpose or objective, as it contravenes DAI/DAS principles.

#### 4.1.5 Handler competence and preparatory training as foundational requirements

Across all PADA exercises the competence of the handler and prior preparation of the dog-handler team are central determinants of welfare components and assessment validity. These factors should not be understood as exercise-specific recommendations but as foundational pre-requisites.

First, handlers must possess a working knowledge of learning theory, including principles of reinforcement, habituation, stress modulation and the ability to accurately identify and interpret canine behavioral and physical welfare indicators and to respond appropriately and instantly. The ability to distinguish between coping, engagement and distress is essential and competence in these domains is therefore not optional but a minimum requirement for ethical participation in any assessment context.

Second, dogs should not be evaluated in a state of naiveté with respect to the types of interactions or environmental conditions they are expected to encounter. Participation in assessments or tests such as PADA presupposes appropriate prior training and preparation like enrichment, conducted using non-aversive, welfare orientated methods. All preparations should follow a process of gradual exposure, ensuring that dogs are introduced progressively to potentially challenging stimuli, under conditions that allow for control, predictability and recovery.

Finally, handler competence and dog preparation are interdependent and must be understood at the level of the specific dog-handler dyad.

#### 4.1.6 Key findings

##### 4.1.6.1 IAHAIO compliance and welfare risk

The majority of PADA exercises do not meet IAHAIO’s welfare provisions across key domains, including voluntary participation, welfare monitoring, proportionality of stimuli, respect for behavioral communication, and provision of recovery opportunities. For example, in social interaction tasks dogs are expected to maintain continuous proximity to unfamiliar persons and tolerate prolonged direct contact (i.e., petting, leaning, hugging, and approach from multiple individuals) without the choice or possibility to increase distance, disengage or retreat, thereby limiting natural canine avoidance or distance-regulating behaviors.

In handling and obedience-based exercises (leash walking, sit, stay) dogs are expected to maintain compliance despite repeated cueing and environmental variability, with restricted scope for initiating alternative behaviors (e.g., Pause, reposition or disengagement) allowing for flexible behavioral adaptation.

Across the examples, the constraint does not lie in the presence of the stimuli per se, but in the restricted opportunity of the dog to modify, terminate or self-regulate its participation in response to internal affective states.

Exercises, such as those involving forced handling or abrupt startle responses, create unnecessary distress and fail to allow the dog to opt-out, violating IAHAIO’s guidelines on voluntary participation and respect for the dog’s communication signals. Only four to five tasks can be considered conditionally compliant—and only if implemented with voluntary engagement and low affective load, i.e., task conditions that are unlikely to induce sustained fear, distress, or high arousal, and that permit the dog to maintain behavioral control and positive or neutral affective states.

In practical interventions opt-out mechanisms can be operationalized through clearly defined and pre-trained signals that allow the dog to indicate that he wants to terminate the interaction or pause participation. Examples include the use of a so-called “exit-target” – here the dog voluntarily approaches and touches a specific object (rubber mat, ball, sock etc.) to indicate disengagement. Also a trained cooperation-signal such as orienting to or settling on a specific mat or blanket will be associated with rest and relief from a task. These signals have been introduced and established during prior training phases under low-arousal conditions, to ensure that they are reliably understood as self-determined opportunities for withdrawal.

Importantly, the use of opt-out mechanisms does not preclude reliable assessment of suitability. On the contrary, the dogs’ willingness to disengage, recover and subsequently re-engage (or opt-in) provides meaningful information about coping capacity, stress tolerance and the quality of the dog- handler relationship. Thus, a welfare-oriented testing framework would integrate opt-in and opt-out signals as part of a broader profile of agency, emotional regulation and adaptive coping, consistent with contemporary welfare standards.

##### 4.1.6.2 Welfare domain impact

Domains 4 and 5 (Behavioral Interactions and Mental State) are consistently affected throughout the exercises. Dogs were placed in situations where they were expected to comply with commands (sit, stay) and deal with unfamiliar or threatening stimuli (sudden movement, noise, ambiguous, potentially threatening, hooded human in dark corridor) which cause emotional stress, potentially traumatizing a dog.

##### 4.1.6.3 Agency and autonomy

The majority of exercises places the dog in passive/reactive roles, where his behavior is dictated by the handler or the evaluator(s) rather than allowing the dog to express natural agency. This holds especially true for exercises that focus on obedience rather than engagement, like forced interactions with unfamiliar objects or people. The lack of competence building or aspirational agency in the exercises restricts the dog’s emotional enrichment and self-determination, which are critical aspects of positive welfare.

### 4.2 Summary of violation levels

As summarized in Figure 1, the cross-exercise analysis highlights recurring welfare, agency, and monitoring concerns across tasks, providing the basis for the detailed findings outlined below. Table 1 contextualizes the specific concerns identified; together, they frame the detailed analysis presented.

High to Very High violation (66, 7 %) comprise the majority of test situations, see Figure 1 and Table 1. Contained within are those with forced proximity, invasive handling, restricted retreat and intense startle stimuli, i.e., conditions creating significant breaches of IAHAIO clauses on avoidance of distress, voluntary participation, and adequate habituation. These exercises furthermore negatively affect Mellor Domains 4 and 5 and result in concomitant agency violations (Špinka, 2019).

Moderate to High violation (27, 8 %) exercises involve novel stimuli presented without choice of distance or time to explore, coerced obedience, ambiguous and frightening human behavior and repeated calling after high stress exercises, see Figure 1 and Table 1. According to Špinka’s (2019) agency levels, these exercises reduce agency to the lowest level. The exercises raise mental-state and affective concerns, creating predictable but probably less severe long-term welfare compromises.

Low to Moderate violations (5, 5%) occur in exercises that are potentially neutral or, if modified, could be positive, such as simple cues/signals or play, see Figure 1 and Table 1. Welfare risks appear low and only develop when dogs are pressured after fatigue or compounded stress with missing acclimatization to new contexts.

## 5 Discussion

Dog-Assisted Interventions/DAS are promoted as mutually beneficial to human and non-human animals. Accumulating evidence, however, raises critical concerns how canine welfare is conceptualized and safeguarded in practice, during assessments and standardized tests (Cortesi et al., 2025; Erichsmeier, 2024; Erichsmeier et al., 2025). Research consistently describes that handlers and even trained observers frequently overlook canine early stress signals (Cortesi et al., 2025; Erichsmeier et al., 2025; Mair et al., 2025; Mariti et al., 2012).

Within this context PADA and similar selection-tools warrant critical scrutiny. When incorporating dogs into interventions indispensable ethical considerations must be considered to protect the welfare of all involved. We must appreciate the critical role that animal welfare plays in enhancing the quality of any DAI/DAS. Given the - mostly anecdotally founded - overemphasis on DAI/DAS outcomes, which may bias handler and participant perception, the potential consequences for the animals involved remain less visible and harder to measure, making appropriate testing for suitability profoundly challenging (Cortesi et al., 2025; Gee and Fine, 2014; McCullough et al., 2021).

Standardized tests are, by definition, designed to measure abilities or traits of individuals using identical tasks and conditions in artificial environments (Kawai et al., 2025; Mair et al., 2025; Richter et al., 2009; Goslin, 1968). Therefore standardized selection tools often lack routine or real-life validity, with behaviors measured not reflecting everyday intelligence, application or skills (Erichsmeier et al., 2025; Harris et al., 2011; Mair, 2025). While formalized criteria aim to ensure consistency across evaluations, they fail to account for the diversity in how dogs (and other animals, human or non-human) react to or in different environments or situations. For instance, a dog that is naturally reserved may not perform well in a test that rewards extroverted, high-energy behavior, even though it may be highly effective and perfectly suited in calmer, more intimate therapy settings (Cortesi et al., 2025; Erichsmeier et al., 2025; Horowitz, 2025).

Cognition varies; an emotional state differs between individuals and across situations and a dog may perform poorly not because he lacks ability but because he feels unsafe or has not yet learned appropriate strategies. Distress generated in testing situations changes behavior, attention, and performance, potentially creating anxiety, fear, frustration and helplessness. Dogs may not understand the purpose of a task or what is expected of them and temporary factors (noise, heat, visual cues) influence results, which cannot be generalized across context and species (Cortesi et al., 2025; Glenk and Foltin, 2021). PADA measures conformity rather than competence, values obedience over cooperation, not reflecting individual skills and abilities. Assessing emotional states during these activities—ensuring that dogs do not experience distress and, ideally, that they enjoy the interaction—is a fundamental aspect of both the selection and the evaluation process prior to a “professional” setting and intervention with recipients (Ameli et al., 2025; de Winkel et al., 2024; Townsend and Gee, 2021; Winkle et al., 2020). By oversimplification of complex behavior, biological differences, cognitive diversity and affective states biased, one-dimensional outcomes result, providing incomplete or inaccurate representations of the tested dogs and of the requirements tested for.

Personality Assessment for Dogs in Animal Assisted Interventions purports to identify dogs with specific personality traits — such as calmness, sociability, and acceptance —, which are expected to make them “safe” for DAI/DAS. Yet these criteria enclose a powerful normative assumption: that good welfare equates to controllability. Dogs are rewarded for emotional subservience and punished, implicitly or explicitly, for spontaneity or avoidance. Explicit punishment involves direct, observable action from the handler intended to correct or stop a behavior, e.g., leash corrections, verbal reprimands or other aversive interventions where dogs experience a contingent negative consequence following a behavior. Implicit punishments refer to processes where the dog is discouraged without overt correction. For example, the handler may withhold social support or expected rewards and create social pressure to maintain compliance. The dog may learn that to express discomfort leads to loss of reinforcement or increased tension, which may suppress communication signals over time.

Treating personality as a fixed diagnostic category risks freezing welfare within a single moment of testing, ignoring the dynamic, context-, age-, and experience-dependent nature of stress responses. Moreover, the very behaviors prized in PADA — tolerance, immobility, neutrality — may indicate behavioral suppression rather than welfare.

Behaviors such as tolerance, emotional neutrality and immobility are often operationally valued within standardized assessments as indicators of behavioral control and task suitability – within this logic they function as markers of compliance and reliability; however, their interpretation in terms of welfare issues is more complex. From a welfare perspective, these behaviors ambiguously reflect affective states, indicating a range of underlying conditions, including effective coping, passive stress responses or behavioral inhibition. Accordingly, any welfare interference must differentiate between immediate affective state, coping strategy and long-term welfare outcomes.

In relation to the rather obedience-structured PADA, behaviors that facilitate human predictability and control may be selectively reinforced, regardless of their underlying emotional basis. From a welfare science perspective this is problematic, because the same outward behavior may arise from fundamentally different internal states. This distinction highlights the central issue raised throughout this manuscript: the potential divergence between behavioral obedience and welfare status.

While PADA may implicitly treat compliant, low-reactivity behavior as indicative of suitability, such interpretation risks associating functional performance with positive emotional states. In contrast, welfare frameworks distinguish between immediate affective states, coping style and longer-term outcomes, like the cumulative impact of experiences over time, all of which may be differently expressed in observable behavior.

There may be a selection bias risk: PADA may favor dogs that endure or tolerate aversive or unpredictable stimuli without overt protest, rather than those that actually enjoy human interaction — an ethical and validity problem for DAI/DAS, as suitability may become equated with subordination. The tests structure privileges human comfort through canine passivity, producing a moral inversion: While DAIs are intended to be empathy driven from the human side, the dog’s own affective states – including distress, engagement, and comfort – are largely unacknowledged, remaining one-directional, and prioritizing human comfort over canine agency.

Standardized tests by definition reject difficile emotional and behavioral diversity of unique dog idiosyncrasies. Diversity is a powerful tool, however, allowing for a broad range of responses, skills, and creative approaches, which can be tailored to meet the specific needs of the recipient and the dog. The distinct personality of the dog helps foster authentic, meaningful connections between dog and recipient, benefiting and enhancing the process, making it a unique and special experience.

Adjusting assessments to recognize and value neurodiversity and manifold characteristics creates a more compassionate and effective approach to DAI/DAS. A one-size-fits-all methodology is not suitable as only canine-adapted evaluations improve the quality of services provided and prioritize the wellbeing of dogs, ensuring a long-term success for all involved (Cortesi et al., 2025).

This welfare audit of all 18 PADA exercises establishes, despite PADAs procedural standardization, a consistent pattern of incompatibility with contemporary standards for ethical evaluations and a substantive distance from positive welfare principles (Fine and Griffin, 2022; Peralta and Fine, 2021; Winkle et al., 2020). Mellor’s model advances welfare assessment beyond the “Five Freedoms” by emphasizing positive mental states and the fulfillment of behavioral needs. PADA, however, narrows this mandate by prioritizing human expectations of therapeutic suitability over canine emotional expression. A dog that complies may appear well-adapted, yet compliance frequently masks stress, learned helplessness and anticipatory anxiety (Seligman, 1973; Boddez et al., 2022; Littlewood et al., 2023). In practice, this creates dogs that appear suitable but their behavior is shaped by conditioning not choice (Horowitz, 2021). Standardized test such as PADA rely on relatively stable behavioral descriptors (e.g., calmness, sociability, non-reactivity) to determine suitability for DAI/DAS. While this approach supports comparability, it also introduces limitations: Behavioral profiles captured in a structured test condition may not adequately reflect context-dependent variables in emotional state and behavior across diverse environments and interactional demands. In this sense, behavioral expression may be shaped by the testing context itself, including novelty, constraint and the absence of choice. As a result, observed compliance may reflect situational adaption rather than stable traits indicative of future performance.

This raises a methodological concern regarding the interpretation of the assessment result. If suitability is primarily inferred from behavior displayed under standardized and potentially non-voluntarily conditions, there is a risk that context-specific responses are overgeneralized as stable characteristics, leading to an over-simplification and may insufficiently capture the dynamic nature of canine behavior relevant to DAI/DAS.

Numerous PADA exercises intentionally evoke fear or startle responses (e.g., screaming, intense knocking, dropped objects, forced handling) thereby contradicting IAHAIO standards and Mellor’s Domains (see Figure 1 and Table 1). Beyond avoiding negative states, Ng et al.’s (2014), Ng’s (2021) welfare optimization framework maintains that ethical assessment should work toward maximizing positive experiences, yet PADA’s procedures are built around eliciting negative affect to measure recovery or tolerance. The reliance on aversive stimuli is scientifically problematic, because negative affect may engulf cognitive processes. Affective neuroscience demonstrates that welfare depends not only on the absence of fear or distress, but on the presence of positive affective states such as curiosity, pleasure, and satisfaction (Casey et al., 2021; Fernandes et al., 2017; Panksepp, 2005). When animals lack opportunities for agency, novelty and control, they may exhibit a passive behavioral pattern of emotional resignation (Erichsmeier et al., 2025; Littlewood et al., 2023). They suppress avoidance signals and may appear “obedient,” yet their behavior may reflect learned helplessness (Seligman, 1973; Boddez et al., 2022), a pattern widely highlighted as a risk in DAI/DAS contexts (Horowitz, 2021; Townsend and Gee, 2021).

To clarify, such behavioral and physiological reactions (startle responses, withdrawal, freezing, increased arousal) primarily reflect immediate affective states and coping response. In isolation these responses do not necessarily indicate persistent welfare impairment. However, they do become relevant from a welfare perspective, when considered in relation to (a) the intensity and controllability of the stimulus, (b) the dogs’ ability to recover within or between exercises, and (c) how such responses are subsequently interpreted for selection decisions.

The welfare concern lies not in the transient exposure itself, but in the potential extrapolation from short-term, context-dependent reactions to broader assumptions about suitability and behavioral resilience across future working contexts.

Accordingly, the argument is grounded in distinction between immediate affective response, short term coping, and recovery process as well as long-term welfare issues, shaped by repeated exposure and subsequent negative working conditions.

Most importantly, across all exercises, PADA provides minimal guidance on stress detection, mitigation, or structured recovery periods. IAHAIO and other welfare guidelines emphasize the need for trained observers, clear stop-rules, and pro-active protection of the animals involved. Yet PADA’s statements such as “if the dog is too stressed, the evaluator may turn away” lack operational definitions and risk normalizing subtle distress signals—panting, freezing, displacement behaviors—which evidence suggests can precede more serious welfare compromise (Ng, 2013; Horowitz, 2021; Townsend and Gee, 2021). A high-quality DAI/DAS depends on the responsibility and competence of the handler. Although the focus is often placed on the dog’s behavior, the most important factor is the handler’s competence. It is the handler who must evaluate each setting and situation and determine how to adapt the environment according to the dog’s needs (Horowitz, 2021).

Fine and Griffin (2022) emphasize that ethical practice requires proactive welfare monitoring, not reactive or subjective judgments. Along these lines, Ng et al. (2024) points out that animal participation must remain voluntary and any behavioral signals in a setting require continuous, context-sensitive interpretation of the handler. Ng et al. (2024) posits systematic welfare assessment strategies like individualized planning of settings, environmental management and multimodal stress monitoring to ensure a sense of control, foreseeability, predictability and choice for the animal.

Personality Assessment for Dogs in Animal Assisted Interventions exhibits systematic ethical and scientific limitations when evaluated against contemporary welfare science, IAHAIO’s and the One Welfare framework. Pervasive issue across exercises is restricted agency, with dogs required to comply with human commands, tolerating intrusive interactions, or withstanding startling stimuli without the ability to opt-out (§4.1). Also, a deficiency of stop/termination criteria (§ 2, 3) and the unproportional use of stressors without appropriate justification (§3.4) are noted. These exercises directly contradict Jones’s (2025) “Canine Consent and Agency Model,” which maintains that dogs need the ability to offer, refuse, and withdraw consent to be ethically defensible. Špinka (2019) underscores that animals achieve positive welfare when they maintain control over exposure and are able to modify contingencies through behavior. PADA’s repeated commands (e.g., “come,” “sit”), leash practice and forced proximity tests systematically remove such control. Applying Špinka’s hierarchy, PADA places dogs largely in passive/reactive roles, thereby denying agency.

Agency—the capacity to make choices that affect one’s own experience—is recognized as a central element to positive welfare. Rault et al. (2025), Jones (2025) emphasize that agency is not merely the absence of coercion but the presence of meaningful control and choice enabling animals to initiate, modify, or terminate interactions, engaging in voluntary, self-generated, goal-directed behavior. Within the structured and temporarily limited context of PADA, dogs have restricted opportunities to exercise choice, control, or withdrawal, as task sequences and expected responses are largely pre-defined to ensure standardization and comparability. While the structure is methodologically aligned with the aims of behavioral testing, it necessarily constraints the expression of agency during the test itself and should thus be understood as a feature of the testing context rather than a model for everyday DAS/DAI practice.

This becomes particularly relevant, when assessment results are interpreted as indicators of broader suitability. As outlined above, behaviors such as compliance or tolerance may be shaped by the restrictions of the exercises, where opportunities for disengagement or behavioral modulation are limited.

From an agency-oriented perspective, the ability to regulate one’s own participation – including the option to disengage – is a central component of positive welfare. Integrating structured opt-out mechanisms offers a way to reconcile methodological requirements with welfare aspects.

Importantly, such mechanisms do not undermine assessment reliability but rather strengthen it. Recording when and how a dog chooses to disengage, how quickly it recovers, and whether it voluntarily re-engages provides meaningful information about coping style, emotional regulation, and the quality of the dog-handler relationship.

A welfare-informed refinement of any test or assessment would therefore aim to maintain comparability while explicitly integrating agency, choice and withdrawal as observable and interpretable components of suitability.

To move beyond these limitations, a redesigned PADA should be grounded in voluntary engagement, consent, and emotional reciprocity. We propose (a) voluntary participation and unpenalised withdrawal; furthermore, consent protocols (for the dog) should be implemented. The training ethos needs to be shifted and handlers must be qualified prior to any test in recognizing avoidance/refusal (opt-out) behavior of the dog as legitimate. Cooperation-based interactions should be prioritized over obedience-based reliability; (b) graded exposure to novel stimuli and exploration should be implemented; (c) positive reinforcement, dissociated from obedience, and the design of a rest and choice area to ensure a dog may pause, engage or disengage from sessions; (d) observer-driven welfare safeguards with explicit stop criteria; (e) accommodation of individual diversity (size, mobility, age, learning history), and a redefinition of DAI/DAS professionalism to include play, curiosity, positive affect, teamwork and reliance on the handler to be a safe harbor and a safe base; (f) limits on cumulative stress through mandated recovery intervals; and (g) a safe environmental design that minimizes physical risk. Welfare should be assessed dynamically, incorporate agency and affective indicators not merely compliance metrics.

Integrating these principles would align the protocol with IAHAIO’s guidelines, Spinka’s agency principles and Mellor’s Five Domains, focusing upon the One Welfare orientation, strengthen scientific validity, and reposition the assessment toward evaluating genuine sociability, resilience, self determination and positive affect.

### 5.1 Recommendations

The analysis of PADA exercises revealed a number of deficits, including:

1. Suppressed canine agency: Many exercises restrict the dog’s natural behavior and fail to provide voluntary opt-in or opt-out mechanisms. Modifications may incorporate more opportunities for the dog to exercise its agency, such as allowing the dog to choose whether to engage in specific tasks.
2. Distress-based stimuli: Exercises involving startle responses or forced handling should be reconsidered, as they conflict with positive welfare paradigms. More gradual exposure and predictable conditions could be implemented to minimize emotional stress.
3. Welfare monitoring deficiencies: A lack of welfare-trained observers and structured recovery periods risks cumulative distress. Clear stop rules and recovery protocols may be introduced to safeguard the dog’s welfare during assessments. Individual qualifications of PADA observer’s data are not systematically reported in the available protocol documentation. PADA does not consistently define or specify a requirement for welfare-specific training. The lack of explicit requirements within the PADA framework, specifying that observer’s must have formal uniform training in animal welfare standards, non-aversive training principles, stress recognition or related ethological competencies forms the basis of concern. There appears to be a structural gap in the protocol design, namely that observer competence in welfare monitoring is not operationalized or formally required in a standardized manner, despite being central to valid and ethical assessment outcomes.
4. Handler training: Handlers must be properly trained to recognize their dog’s signs of distress and engage with the dog in a humane and ethical manner. Handlers should be encouraged to prioritize the dog’s welfare over performance outcomes.

### 5.2 The tension between scientific welfare standards and standardized assessments

A central challenge emerging from this analysis is the inherent tension between advancing and addressing contemporary scientific welfare standards and maintaining the psychometric requirements of standardized assessments. Tests such as PADA are designed with the idea to apply reliability, comparability and predictive validity across evaluators and contexts. From this perspective the inclusion of challenging stimuli – such as sudden noise or social pressure – may be justified as a means of assessing coping capacity, emotional regulation or behavioral flexibility.

However, this rational requires careful ethical qualification. Exposure to such stimuli must not be equated with the indiscriminate induction of stress or the suppression of behavioral expression. Contemporary frameworks (such as the IAHAIO guidelines, the Five Domains Model and the Agency-Based welfare science) emphasize that welfare is not merely the ability to tolerate adversity, but the capacity to experience positive states, exercise control and engage voluntarily.

Accordingly, the ethical legitimacy of standardized testing depends on how such challenges are implemented. Stimuli must be graded, predictable, foreseeable, and embedded within a framework that allows for continuous monitoring, immediate withdrawal, and recovery. Crucially, the dog must retain the ability meaningful control over its participation, including to opt-out without penalty. In this sense, the aim of any assessment should shift from measuring passive tolerance toward evaluating adaptive coping within a context of agency, relational support and safety. Only under these conditions can standardized procedures be reconciled with contemporary welfare science and the ethical imperatives of DAI/DAS.

### 5.3 Limitations

While the study is using a clearly articulated framework (IAHAIO guidelines, Špinka’s hierarchy, Mellor’s Five Domains) and systematic analysis, I acknowledge that it is interpretative in nature and relies on publicly available documents and literature. As such, findings are limited to the completeness and accuracy of these sources and by the potential contextual factors in exercise implementation that were not observable. These limitations may affect the generalizability of the conclusions herein. A statement on author positionality is hereby added, clarifying that the analysis reflects the expertise in theory and practice in animal welfare, DAI/DAS ethics, behavior, and scientific background and that the interpretations are informed by this background. It may be noted, that while this expertise strengthens the ability to identify welfare concerns and ethical issues it may also shape the focus and emphasis of the analysis. Despite the interpretative nature of this work established welfare frameworks have been systematically applied, multiple sources have been cross-referenced and results are presented in a structured and transparent manner, enhancing the credibility and reproducability of the findings.

## 6 Conclusion

The PADA assessment system, while widely used, reveals significant welfare risks due to its focus on compliance over agency and its use of distressing stimuli. By integrating recommendations that prioritize canine agency, voluntary participation, and emotional enrichment, the PADA system may be better aligned with contemporary welfare frameworks and ethical standards, ensuring the wellbeing of the dogs and the success of Dog-Assisted Interventions.

## Footnotes

1
https://www.pada-icofa.com/

2
https://pada.zwierzetaludziom.pl/
