# Supplementary material for: Agency denied: PADA and the perils of paternalistic dog care
Source: Front Psychol. 2026 Jul 9;17:1767922. doi: 10.3389/fpsyg.2026.1767922 (PMC13391819; doi:10.3389/fpsyg.2026.1767922)
Supplement: Supplementary file 1 [file Supplementary_file_1.docx]

**Supplemental Materials**

## Key IAHAIO clauses referenced

- **§2.3** Competence of observers (welfare-trained monitoring & stop/opt-in/opt-out rules)
- **§2.5** Welfare before standardization (tests that cause anxiety for reliability)
- **§3.2** Freedom from distress (avoid placing dogs in threatening/aversive/potentially traumatizing situations unnecessarily)
- **§3.4** Proportionality & necessity (use of stressors only if essential and proportionate to the purpose)
- **§3.5** Respect for species-specific signals & individual differences
- **§4.1** Voluntary participation / right to withdraw
- **§4.4** Habituation & preparation / predictability
- **§4.5** Positive interactions and positive affect emphasis
- **§4.6** Debrief/recovery and post-event welfare
  1. **Evaluated Dimensions,** Špinka, 2019

3. Agency Level Classification (Špinka, 2019) — every exercise was classified according to the dominant agency level the dog was allowed to express:

(1) Passive/reactive agency – responses constrained by human control.

(2) Action-driven agency – limited scope for behavioral expression.

(3) Competence-building agency – opportunities for learning and mastery.

(4) Aspirational agency – proactive engagement, intrinsic motivation, and emotional enrichment.
